# Modeling Charge Transfer Reactions by Hopping between Electronic Ground State Minima: Application to Hole Transfer between DNA Bases

**DOI:** 10.3390/molecules27217408

**Published:** 2022-11-01

**Authors:** Alessandro Nicola Nardi, Marco D’Abramo, Andrea Amadei

**Affiliations:** 1 Department of Chemistry, Sapienza University, 00185 Rome, Italy; 2 Department of Chemical and Technological Sciences, Tor Vergata University, 00133 Rome, Italy

**Keywords:** charge transfer, DNA, theoretical–computational chemistry

## Abstract

In this paper, we extend the previously described general model for charge transfer reactions, introducing specific changes to treat the hopping between energy minima of the electronic ground state (i.e., transitions between the corresponding vibrational ground states). We applied the theoretical–computational model to the charge transfer reactions in DNA molecules which still represent a challenge for a rational full understanding of their mechanism. Results show that the presented model can provide a valid, relatively simple, approach to quantitatively study such reactions shedding light on several important aspects of the reaction mechanism.

## 1. Introduction

The charge migrations through DNA represent a very interesting subject because they are relevant in different areas, ranging from biochemistry to technology. In fact, the understanding of the charge fluxes occurring within DNA can help to describe, among others, cell oxidative damage processes as well as to design DNA-based sensors [1,2]. Therefore, this subject has been extensively studied by both experimental and theoretical–computational techniques. Although so far the high complexity of the system has partially hindered a complete characterization of the charge transfer processes in DNA, recent technical advances in spectroscopic instrumentation contributed to enhance our understanding of the kinetics of such phenomena. That is, starting from the first direct measurement of the photoinduced hole transport in DNA by time-resolved spectroscopy [3], several experimental works have contributed to shed light on this subject [4,5,6,7,8,9,10,11]. In particular, DNA hole transfer kinetics through π-stack arrays have been proved to depend not only from the redox potential of the single nucleobases but also on the DNA specific sequence and conformation [12] as revealed by means of time-resolved spectroscopic data. From a mechanistic viewpoint, charge migration along DNA is explained by super exchange and hole hopping [13,14,15,16]. In the former, the hole migration occurs via direct tunneling from the charge donor to the charge acceptor, as observed in relatively short DNA hairpins and sequences [17,18,19]. On the other hand, in longer hairpins and sequences [20,21,22] within G-rich double-stranded DNA, the charge migration is achieved by subsequent charge hopping steps where Guanines, the nucleobases with the lowest oxidation potentials, act as charge carriers thus indicating a charge flux mechanism based on subsequent Guanines hole hopping steps, where at each step the super exchange mechanism provides the charge transfer between the subsequent Guanines, regardless of the in between base bridge present. However, the possible charge delocalization along DNA has prompted the idea that formation of polarons [23,24], i.e., radical cations where the excess of positive charge is shared by nucleobases, could be relevant in DNA charge transfer [16,25]. In recent papers it has been hypothesized that weak fluctuations around the DNA equilibrium structure can induce charge delocalized bridge states from an initial localized charge donor state in a single thermally activated step, i.e., quantum unfurling [26]. This mechanism should be dominating in ordered bridges, but local uncorrelated fluctuations could lead to charge localized electronic bridge states, resulting in a base to base hopping-like mechanism [27]. The role of fluctuations has been also invoked in the flickering resonance mechanism, suggesting that molecular structure/medium thermal fluctuations can produce transient energy degeneracy among multiple CT sites, possibly explaining the exponential rate decay with the bridge length and coexisting with other mechanisms (super exchange, base to base hopping, etc.) [28]. The need of a detailed explanation of the charge transfer along DNA, still representing a matter of debate [29,30], stimulated several theoretical–computational groups aiming to rationalize the available experimental data. Unfortunately, in-silico approaches are still limited by the necessity to provide an accurate description of the electronic properties of the donor and acceptor over an extended sampling of the DNA molecule structures, in particular including the effect of a realistic dynamical perturbing environment, typically disregarded in the available models, resulting in fluctuating donor-acceptor quantum properties. To address the charge transfer kinetics in DNA by means of a general theoretical–computational approach specifically including the dynamical environment perturbation and DNA fluctuations, we report here our modelling of the super exchange charge transfer within a single step hole hopping between Guanine bases, as occurring in different double-stranded DNA molecules, and compare our results with the available experimental data [8]. More specifically, in the Theory section we derive a general model to treat charge transfer (CT) reactions corresponding to the hopping (i.e., vibrational state tunnelling) between different minima of the electronic ground state (the relevant case for DNA base CT, according to our data) and in the results section we characterize in detail the CT process as obtained for different lengths and types of the bridge separating the Guanine bases involved in the reaction.

## 2. Theory

### 2.1. General Considerations

In a previous paper [31] we described, in detail, the general model to treat CT reactions in complex systems. However, in that paper we specifically considered CT reactions involving diabatic states defined by vibronic states corresponding to different electronic states, disregarding the case of diabatic states involving the same electronic state (e.g., defined via the vibrational ground states of different energy minima of the electronic ground state). Although such a condition (the case presently studied) can be still treated via the general model we presented, specific changes and discussions are necessary. First we need to introduce a few basic definitions/assumptions to be used:

- We assume that we can divide the simulated system into the QC, the subpart where the reaction occurs thus requiring a quatum mechanical treatment, and its atomic-molecular environment which we model as a semiclassical atomistic subsystem; moreover, we consider the environment subsystem internal energy as invariant for each QC quantum state transition and thus disregarded when evaluating the energy change involved in the reaction.
- We define the QC adiabatic states via the QC vibronic eigenstates (adiabatic vibronic states) possibly involving the nuclei-electron coupling (non-adiabatic coupling) which can be non-negligible when degenerate or quasi-degenerate electronic states are concerned.
- As QC diabatic states we define the vibronic Born–Oppenheimer eigenstates of the perturbed QC corresponding to the electron distribution constrained to be fixed in either the reactant (*R*) or the product (*P*) chemical state, according to the *R* and *P* chemical species involved in the reaction. Moreover, we assume the non-adiabatic coupling as providing approximately negligible effects when using the diabatic states basis set.
- We assume each possible reactive event as properly described by only two QC adiabatic states. We always consider such adiabatic states as virtually indistinguishable from the corresponding diabatic ones except within a tiny perturbation region (the transition region, TR), including the crossing of the diabatic energy surfaces, where the reaction event occurs. We also always assume that outside the transition region the reaction statistical ensemble (the ensemble of the reactive trajectories) be fully equivalent to the one provided by reactive trajectories with the QC in either the *R* or *P* QC diabatic state: i.e., although within a single reactive trajectory the QC can be in a linear combination of the two diabatic states, the statistics can be conceived as given by QC sub-populations in either the *R* or *P* state. Therefore, for each trajectory of the reactive ensemble corresponding to e.g., the R→P transition, the QC can be thought to be in the *R* state when entering the TR and in either the *R* or *P* state when leaving the TR and hence the reaction dynamics within the reactive ensemble (the *R* to *P* inter-conversion) can be modeled by the usual equations of chemical kinetics (i.e., we assume the QC-environment system as a dissipative quantum system within the Markoff approximation [32,33,34]). Only when considering the reactive trajectories within the TR’s we need to explicitly account for the quantum dynamics of each reactive trajectory as within the TR the Markoff approximation is typically inapplicable and the QC quantum dynamics usually involves mixing of the diabatic/adiabatic states.
- We assume that, given its tiny dimension, each TR traversing be fast enough to avoid any relevant change of the QC semiclassical coordinates and thus of the diabatic states, which can be then considered as time-independent within the whole reaction event (i.e., the TR crossing) with a virtually constant coupling term and linear time-dependent diabatic energies. By also assuming that such a short traversing time may correspond to a large (virtually infinite) relaxation time for the QC dynamical quantum state, we can use the Landau–Zener approach [35,36] to model the diabatic/adiabatic behaviour of the reaction event, i.e. the probability for the *R* and *P* diabatic states as obtained by the QC quantum dynamics when emerging from the TR.

**Figure 1:**
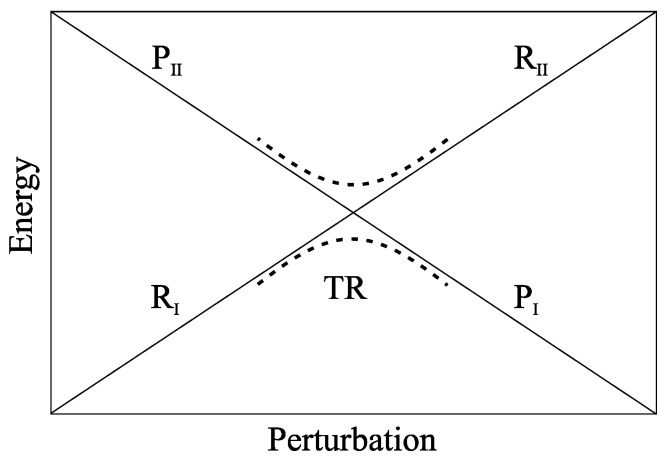
Schematic description of the diabatic (**solid lines**) and adiabatic (**dashed lines**) energy surfaces within the energy vs. perturbation plane.

From Figure 1 and according to our previous paper [31], defining with *A* and *B* the *I* and II adiabatic surface index, respectively, the rate equations for the R→P reaction when considering $R_{A}$ the initial reactant state and $R_{B}$ within a steady state condition, are

(1) $$\begin{array}{ccc} \dot{\left[R_{A}\right]} & ≅ & -\alpha \left(2-\alpha \right)K_{R_{A}}\left[R_{A}\right] \end{array}$$

(2) $$\begin{array}{ccc} \dot{\left[P\right]} & ≅ & -\dot{\left[R_{A}\right]}≅\alpha \left(2-\alpha \right)K_{R_{A}}\left[R_{A}\right]\; \end{array}$$

where $K_{R_{A}}$ is the reaction kinetic constant and α is the transmission coefficient as obtained by the TR crossings via the Landau-Zener approximation (see the Appendix A for more details), providing

(3) $$\begin{array}{ccc} \left[R_{A}\right] & ≅ & {\left[R_{A}\right]}_{0}\;e^{-\alpha \left(2-\alpha \right)K_{R_{A}}t} \end{array}$$

(4) $$\begin{array}{ccc} \left[P\right] & ≅ & {\left[R_{A}\right]}_{0}\left[1-e^{-\alpha \left(2-\alpha \right)K_{R_{A}}t}\right]\; \end{array}$$

with ${\left[R_{A}\right]}_{0}$ the $R_{A}$ concentration at the beginning of the $R_{B}$ steady state condition (see the Appendix A). In the following we will always consider the charge transfer reaction as occurring according to Equations (1)–(4) (i.e., $R_{A}$ is the initial reactant state).

### 2.2. The Gaussian Approximation

**Figure A1:**
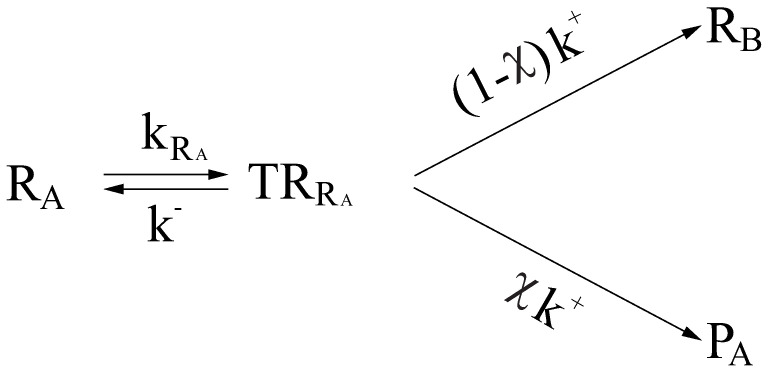
Reaction scheme for the $R_{A}\to P$ transition as occurring within a single TR reaction (see text). $TR_{R_{A}}$ represents the TR ensemble provided by the reactive trajectories entering the transition region from $R_{A}$. A similar scheme can be used for the $R_{B}\to P$ transition.

Equations (1)–(4) provide the link between the chemical kinetics model and the data obtained by the computational simulation. In fact, by means of a large set of proper MD simulations in combination with quantum calculations it is possible, for a given *R* and *P* diabatic state couple, to evaluate the distribution of the time-lengths needed by the QC for reaching the diabatic energy surface crossings in the reactant ensemble and thus to reconstruct the kinetic trace of $\left[R_{A}\right]$ providing the corresponding kinetic constant $K_{R_{A}}$, as shown in previous papers [31,37,38,39,40]. Moreover, by means of the Landau–Zener approximation, from the ensemble of the diabatic surface crossings as obtained by the reactive trajectories we can estimate α=〈χ〉 by evaluating the adiabatic fraction χ at each crossing and then averaging over all the crossings. In the unfortunate case the kinetics is too slow to obtain a proper crossing sampling from MD simulations (as for the CT reactions investigated in this paper), we can still reasonably evaluate $K_{R_{A}}$ assuming an approximately Gaussian behavior for the diabatic energy difference (the transition energy ΔU) fluctuations around the transition energy mode value (i.e., the transition energy corresponding to the probability distribution maximum) $\Delta U_{R_{A}}≅{\langle \Delta U\rangle }_{R_{A}}$, with such a Gaussian range including the diabatic energy crossing, i.e., ΔU=0 (note that the $R_{A}$ subscript of the angle brackets indicates that averaging is performed in the $R_{A}$ equilibrium ensemble). In fact, considering that within each TR we have a virtually unidirectional flux ($k^{-}≅0$, see the Appendix A) with an exit rate constant $k^{+}≅v_{cr}/\delta$ where δ is the TR transition energy range that we assume virtually identical for all the transition regions and $v_{cr}$ the norm of the crossing velocity, we obtain [41] that at equilibrium the corresponding rate constants (i.e., the rate constants for the TR forward and backward fluxes) must be virtually identical and well approximated by $v_{cr}/\left(2\delta \right)$, and hence

(5) $$\begin{array}{ccc} K_{R_{A}} & ≅ & {\langle k_{R_{A}}\rangle }_{A}≅\frac{{\langle k_{R_{A}}\rangle }_{A}}{{\langle v_{cr}\rangle }_{TR}/\left(2\delta \right)}\;\frac{{\langle v_{cr}\rangle }_{TR}}{2\delta }≅\frac{Q_{TR}}{Q_{R_{A}}}\;\frac{{\langle v_{cr}\rangle }_{TR}}{2\delta }\\ & = & \frac{Q_{TR}}{Q_{R_{A}}}\;\frac{\langle v\rangle }{2\delta }\; \end{array}$$

where $k_{R_{A}}$ is the kinetic constant for the $R_{A}\to P$ transition as occurring within a single TR reaction (i.e., a reaction process defined by crossing events with an almost identical χ value) as shown by Figure A1 in the Appendix A, the subscript *A* of the angle brackets means that averaging was obtained over all the TR reactions within the $R_{A}\to P$ reactive ensemble, $\langle v\rangle ={\langle v\rangle }_{R_{A}}={\langle v_{cr}\rangle }_{TR}$ (with *v* the norm of the transition energy time derivative and the TR subscript indicating averaging within the equilibrium full TR ensemble), $Q_{TR}$ and $Q_{R_{A}}$ are the canonical partition functions of the TR and $R_{A}$ chemical state, respectively, and we assumed

(6) $${\langle k_{R_{A}}\rangle }_{A}≅{\langle k_{R_{A}}\rangle }_{R_{A}}$$

providing

(7) $$\frac{{\langle k_{R_{A}}\rangle }_{A}}{{\langle v_{cr}\rangle }_{TR}/\left(2\delta \right)}≅\frac{{\langle k_{R_{A}}\rangle }_{R_{A}}}{{\langle v_{cr}\rangle }_{TR}/\left(2\delta \right)}≅\frac{Q_{TR}}{Q_{R_{A}}}$$

Note that the chemical states refer always to a single QC with thus the corresponding canonical partition functions obtained for the QC-environment system when constraining the QC into a given chemical state.

Equation (6) simply means that the $R_{A}$ sub-populations involved into the different single TR reactions are in fast inter-conversion, allowing us to consider their distribution within the reactive trajectory ensemble used (i.e., the $R_{A}\to P$ ensemble) as a stationary pre-equilibrium distribution virtually identical to the corresponding reactant full equilibrium one. We can express the partition function $Q_{R_{A}}$ via the reactant Landau free energy $A_{R}$ defined by [42]

(8) $$A_{R}\left(\Delta U\right)=-k_{B}T\ln Q_{R}\left(\Delta U\right)$$

with $Q_{R}\left(\Delta U\right)$ the reactant partition function density providing

(9) $$Q_{R_{A}}=\int_{\delta /2}^{\infty }Q_{R}\left(\Delta U\right)d\Delta U$$

and hence the equilibrium probability density for the $R_{A}$ ensemble ($\rho_{R_{A}}$) is obtained via

(10) $$\begin{array}{ccc} \rho_{R_{A}}\left(\Delta U\right) & = & \frac{Q_{R}\left(\Delta U\right)}{Q_{R_{A}}}=\frac{e^{-A_{R}\left(\Delta U\right)/\left(k_{B}T\right)}}{Q_{R_{A}}} \end{array}$$

(11) $$\begin{array}{ccc} & & \frac{\delta }{2}\leq \Delta U\leq \infty \end{array}$$

Note that $\frac{\delta }{2}\leq \Delta U\leq \infty$ is the transition energy range defining the $R_{A}$ chemical state.

Once assuming Gaussian fluctuations for the transition energy within a large range around $\Delta U_{R_{A}}$ with then

(12) $$\Delta U_{R_{A}}≅{\langle \Delta U\rangle }_{R_{A}}$$

we can introduce the $R_{A}$ quadratic Landau free energy ($A_{R_{A}}$) approximating the reactant Landau free energy $A_{R}$ within the proper Gaussian range

(13) $$A_{R_{A}}\left(\Delta U\right)=A_{R}\left(\Delta U_{R_{A}}\right)+\frac{k_{B}T}{2\sigma_{R_{A}}^{2}}{\left(\Delta U-\Delta U_{R_{A}}\right)}^{2}$$

where $k_{B}$ is the Boltzmann constant, *T* is the absolute temperature, $\sigma_{R_{A}}^{2}$ is the transition energy variance for the $R_{A}$ equilibrium ensemble, and

(14) $$A_{R}\left(\Delta U_{R_{A}}\right)≅A_{R}\left({\langle \Delta U\rangle }_{R_{A}}\right)$$

is the corresponding minimum of the Landau free energy. From the last equations, assuming the Gaussian approximation valid within the whole $R_{A}$ range with $|\Delta U_{R_{A}}|/\sigma_{R_{A}}>3$, we can write

(15) $$\begin{array}{ccc} Q_{R_{A}} & = & \int_{\delta /2}^{\infty }e^{-A_{R}\left(\Delta U\right)/\left(k_{B}T\right)}d\Delta U≅\int_{-\infty }^{\infty }e^{-A_{R_{A}}\left(\Delta U\right)/\left(k_{B}T\right)}d\Delta U\\ & = & e^{-A_{R}\left(\Delta U_{R_{A}}\right)/\left(k_{B}T\right)}\sqrt{2\pi \sigma_{R_{A}}^{2}} \end{array}$$

For the TR chemical state (i.e., the chemical state defined by all the TR’s), given the tiny transition energy range δ, we can assume its Landau free energy $A_{TR}$ being virtually constant with $A_{TR}=A_{R}\left(0\right)≅A_{R_{A}}\left(0\right)$, and hence

(16) $$\begin{array}{ccc} Q_{TR} & ≅ & \int_{-\delta /2}^{\delta /2}e^{-A_{R}\left(\Delta U\right)/\left(k_{B}T\right)}d\Delta U≅e^{-A_{TR}/\left(k_{B}T\right)}\delta \\ & ≅ & e^{-A_{R_{A}}\left(0\right)/\left(k_{B}T\right)}\delta \end{array}$$

From Equations (15) and (16) we readily obtain

(17) $$\frac{Q_{TR}}{Q_{R_{A}}}≅\frac{e^{-\Delta A_{R_{A}}^{†}/\left(k_{B}T\right)}\delta }{\sqrt{2\pi \sigma_{R_{A}}^{2}}}$$

with (see Equations (12) and (13))

(18) $$\begin{array}{ccc} \Delta A_{R_{A}}^{†} & = & A_{R}\left(0\right)-A_{R}\left(\Delta U_{R_{A}}\right)\\ & ≅ & A_{R_{A}}\left(0\right)-A_{R}\left(\Delta U_{R_{A}}\right)≅\frac{k_{B}T}{2\sigma_{R_{A}}^{2}}{\langle \Delta U\rangle }_{R_{A}}^{2} \end{array}$$

providing, using Equation (5),

(19) $$K_{R_{A}}≅\frac{e^{-\Delta A_{R_{A}}^{†}/\left(k_{B}T\right)}\delta }{\sqrt{2\pi \sigma_{R_{A}}^{2}}}\;\frac{\langle v\rangle }{2\delta }≅\frac{e^{-{\langle \Delta U\rangle }_{R_{A}}^{2}/\left(2\sigma_{R_{A}}^{2}\right)}}{\sqrt{2\pi \sigma_{R_{A}}^{2}}}\;\frac{\langle v\rangle }{2}\;$$

The last equation furnish a typically good approximation for CT processes where the crossing is not too far from the Landau free energy minimum, thus ensuring that the quadratic approximation for modeling the activation Landau free energy ($\Delta A_{R_{A}}^{†}$) is reasonably accurate, although still providing a large Gaussian range (a typically good criterion is $3<|\Delta U_{R_{A}}|/\sigma_{R_{A}}<10$).

### 2.3. The Diabatic States

In the present case we consider CT reactions involving the diabatic energy crossings due to two vibrational states of the electronic ground state, with hence each reaction event essentially equivalent to the hopping between different energy minima (i.e., tunnelling between the corresponding vibrational ground states). Defining such electronic ground state minima as *R* and *P*, providing the two different charge distributions, we can define the corresponding QC *local* vibrational ground eigenstates (in the coordinate representation) via

(20) $$\begin{array}{ccc} \phi_{R}\left(\xi ,\beta \right) & ≅ & \Pi_{l}\phi_{R,l}\left(\beta_{l}\right) \end{array}$$

(21) $$\begin{array}{ccc} \phi_{P}\left(\xi ,\beta \right) & ≅ & \Pi_{l}\phi_{P,l}\left(\beta_{l}\right) \end{array}$$

where ξ,β are the QC nuclear (internal) semiclassical and quantum (harmonic) mode coordinates (defined by the mass weighted Hessian eigenvectors), respectively, and $\phi_{R,l},\phi_{P,l}$ are the (harmonic) *l*th quantum mode vibrational ground states for the *R* and *P* minima. Note that we assume that both minima can be well described by the same harmonic modes and frequencies, differing only for their position $\xi_{R},\beta_{R}$ and $\xi_{P},\beta_{P}$ and energy value (i.e., $\phi_{R,l}$ and $\phi_{P,l}$, approximately independent of the ξ coordinates, only differ for the $\beta_{l}$ minimum energy position: $\beta_{l,R}$ or $\beta_{l,P}$), always considering the roto-translational coordinates as semiclassical degrees of freedom. It is worth to remark that such an assumption, providing Equations (20) and (21), is typically a good approximation when $\xi_{R}\approx \xi_{P}$, possibly becoming inaccurate when a large variation of the semiclassical coordinates is involved in the two energy minima (note that the thermal energy of the system can be used for discriminating between semiclassical and quantum modes, with the quantum modes being those corresponding to an energy gap higher than the thermal energy). Moreover, dealing only with the electronic ground state we always disregard any spin effect on the Hamiltonian operator and hence on the *local* vibrational eigenstates. In order to obtain two proper diabatic states for the CT reaction (essentially the transition from the *R* to *P* minimum) we only have to search for two linear combinations of $\phi_{R}$ and $\phi_{P}$ providing two orthonormal diabatic states $\eta_{R}$ and $\eta_{P}$ as closest as possible to $\phi_{R}$ and $\phi_{P}$, respectively. Considering that the harmonic vibrational eigenstates are real wavefunctions, we obtain

(22) $$\begin{array}{ccc} \eta_{R} & = & c_{1}\phi_{R}-c_{2}\phi_{P} \end{array}$$

(23) $$\begin{array}{ccc} \eta_{P} & = & -c_{2}\phi_{R}+c_{1}\phi_{P} \end{array}$$

(24) $$\begin{array}{ccc} c_{1}^{2}+c_{2}^{2} & = & \frac{1}{1-{\langle \phi_{P}|\phi_{R}\rangle }^{2}} \end{array}$$

(25) $$\begin{array}{ccc} 2c_{1}c_{2} & = & \frac{\langle \phi_{P}|\phi_{R}\rangle }{1-{\langle \phi_{P}|\phi_{R}\rangle }^{2}}=\left(c_{1}^{2}+c_{2}^{2}\right)\langle \phi_{P}|\phi_{R}\rangle \end{array}$$

where $c_{1},c_{2}$ are real coefficients,

(26) $$\begin{array}{ccc} \langle \phi_{P}|\phi_{R}\rangle & = & \Pi_{l}\langle \phi_{P,l}|\phi_{R,l}\rangle \\ & = & \Pi_{l}\langle \phi_{R,l}|\phi_{P,l}\rangle =\langle \phi_{R}|\phi_{P}\rangle \end{array}$$

is the Hermitian product of the (real) *local* vibrational eigenstates (i.e., in the coordinate representation the integral of their algebraic product over the β coordinates) with ${\langle \phi_{P}|\phi_{R}\rangle }^{2}\approx 0$ and thus $c_{1}^{2}\approx 1$$c_{2}^{2}\approx 0$. In order to obtain the CT kinetics we need to evaluate the diabatic energy difference $H_{P,P}-H_{R,R}$ (i.e., the transition energy ΔU) to identify the diabatic energy crossings (i.e., the conical intersections) and the corresponding Hamiltonian coupling $H_{R,P}$ necessary for obtaining the *transmission coefficient* α

(27) $$\begin{array}{ccc} H_{P,P}-H_{R,R} & = & \langle \eta_{P}|\hat{H}|\eta_{P}\rangle -\langle \eta_{R}|\hat{H}|\eta_{R}\rangle \\ & = & \left(c_{1}^{2}-c_{2}^{2}\right)[\langle \phi_{P}|\hat{H}|\phi_{P}\rangle -\langle \phi_{R}|\hat{H}|\phi_{R}\rangle ]\\ & ≅ & \left(c_{1}^{2}-c_{2}^{2}\right)\left[U_{e}\left(\xi_{P},\beta_{P}\right)-U_{e}\left(\xi_{R},\beta_{R}\right)\right]\\ & \approx & U_{e}\left(\xi_{P},\beta_{P}\right)-U_{e}\left(\xi_{R},\beta_{R}\right) \end{array}$$

(28) $$\begin{array}{ccc} H_{R,P} & = & H_{P,R}=\langle \eta_{P}|\hat{H}|\eta_{R}\rangle \\ & = & -c_{1}c_{2}[\langle \phi_{R}|\hat{H}|\phi_{R}\rangle +\langle \phi_{P}|\hat{H}|\phi_{P}\rangle ]+c_{2}^{2}\langle \phi_{R}|\hat{H}|\phi_{P}\rangle +c_{1}^{2}\langle \phi_{P}|\hat{H}|\phi_{R}\rangle \\ & = & -c_{1}c_{2}[\langle \phi_{R}|\hat{H}|\phi_{R}\rangle +\langle \phi_{P}|\hat{H}|\phi_{P}\rangle ]+\left(c_{1}^{2}+c_{2}^{2}\right)\langle \phi_{P}|\hat{H}|\phi_{R}\rangle \end{array}$$

where $\hat{H}≅U_{e}+{\hat{K}}_{\beta }+K_{\xi }$ is the Born–Oppenheimer QC (vibrational) Hamiltonian operator (including the QC-environment interaction) with $U_{e}$ the electronic ground state energy, ${\hat{K}}_{\beta }$ the kinetic energy operator of the nuclear quantum mode coordinates β, $K_{\xi }$ the classical kinetic energy of the semiclassical mode coordinates ξ, and we used $\langle \phi_{P}|\hat{H}|\phi_{P}\rangle -\langle \phi_{R}|\hat{H}|\phi_{R}\rangle ≅U_{e}\left(\xi_{P},\beta_{P}\right)-U_{e}\left(\xi_{R},\beta_{R}\right)$ and $\langle \phi_{R}|\hat{H}|\phi_{P}\rangle =\langle \phi_{P}|\hat{H}|\phi_{R}\rangle$ as following from the fact that $\phi_{R},\phi_{P}$ are real wavefunctions differing only for their minimum energy position. Note that since ξ,β are mass weighted coordinates, we necessarily have ${\hat{K}}_{\beta }=\sum_{l}{\hat{\pi }}_{\beta_{l}}^{2}/2$ and $K_{\xi }=\sum_{j}\pi_{\xi_{j}}^{2}/2$ with ${\hat{\pi }}_{\beta_{l}}$ and $\pi_{\xi_{j}}$ the conjugated momentum operators and classical conjugated momenta of the β and ξ coordinates, respectively. It is also worth noting that the use in Equations (27) and (28) of the Born–Oppenheimer vibrational Hamiltonian, disregarding any (electronic) non-adiabatic coupling, means that we are assuming negligible non-adiabatic coupling for Born–Oppenheimer diabatic states involving the same electronic state.

From Equation (27) it is evident that at each diabatic energy crossing we must have $U_{e}\left(\xi_{P},\beta_{P}\right)-U_{e}\left(\xi_{R},\beta_{R}\right)\approx \langle \phi_{P}|\hat{H}|\phi_{P}\rangle -\langle \phi_{R}|\hat{H}|\phi_{R}\rangle =0$ and thus (using also Equations (24) and (25)) we obtain for the coupling term at the crossing

(29) $$\begin{array}{ccc} H_{R,P}=H_{P,R} & = & -2c_{1}c_{2}\langle \phi_{R}|\hat{H}|\phi_{R}\rangle +\left(c_{1}^{2}+c_{2}^{2}\right)\langle \phi_{P}|\hat{H}|\phi_{R}\rangle \\ & = & \left(c_{1}^{2}+c_{2}^{2}\right)[\langle \phi_{P}|\hat{H}|\phi_{R}\rangle -\langle \phi_{P}|\phi_{R}\rangle \langle \phi_{R}|\hat{H}|\phi_{R}\rangle ]\\ & \approx & \langle \phi_{P}|\hat{H}|\phi_{R}\rangle -\langle \phi_{P}|\phi_{R}\rangle \langle \phi_{R}|\hat{H}|\phi_{R}\rangle \end{array}$$

We can express the Hamiltonian operator via the approximation of independent quantum mode terms (i.e., we disregard anharmonic mode coupling and quantum and semiclassical subspace mixing)

(30) $$\hat{H}\left(\xi ,\beta \right)≅U_{e}\left(\xi_{R},\beta_{R}\right)+\sum_{l}\left[{\hat{K}}_{\beta_{l}}+\Delta U_{e}\left(\xi_{R},\beta_{R}^{\prime },\beta_{l}\right)\right]+K_{\xi }+\Delta U_{e}\left(\xi ,\beta_{R}\right)$$

with ${\hat{K}}_{\beta_{l}}={\hat{\pi }}_{\beta_{l}}^{2}/2$ the lth quantum mode kinetic energy operator, $\Delta U_{e}\left(\xi_{R},\beta_{R}^{\prime },\beta_{l}\right)$ the electronic energy change, with respect to the *R* energy minimum, as obtained moving along the $\beta_{l}$ mode coordinate only ($\beta_{R}^{\prime }$ corresponds to all the other quantum coordinates fixed at their minimum energy position) and $\Delta U_{e}\left(\xi ,\beta_{R}\right)$ the electronic energy change, with respect to the *R* energy minimum, as obtained moving only the semiclassical mode coordinates. By using Equation (30) into Equation (29) we then obtain

(31) $$\begin{array}{ccc} H_{R,P}=H_{P,R} & \approx & \langle \phi_{P}|\phi_{R}\rangle \sum_{l}\{\frac{\langle \phi_{P,l}|\Delta U_{e}\left(\xi_{R},\beta_{R}^{\prime },\beta_{l}\right)-\Delta U_{R,l}\left(\beta_{l}\right)|\phi_{R,l}\rangle }{\langle \phi_{P,l}|\phi_{R,l}\rangle }\\ & - & \langle \phi_{R,l}|\Delta U_{e}\left(\xi_{R},\beta_{R}^{\prime },\beta_{l}\right)-\Delta U_{R,l}\left(\beta_{l}\right)|\phi_{R,l}\rangle \} \end{array}$$

where $\Delta U_{R,l}\left(\beta_{l}\right)$ is the electronic energy change, with respect to the *R* energy minimum, as provided by the *R* purely harmonic behavior of the lth quantum mode (with $\Delta U_{P,l}$ we can define the electronic energy change from the *P* energy minimum, as provided by the *P* purely harmonic behavior of the lth quantum mode). Finally, it is worth to remark that we assume the *R* and *P* minima as sharing the same quantum modes and frequencies.

### 2.4. Practical Strategy

In order to obtain a reasonable evaluation of the transition energy and related R,P coupling, according to the previous subsection, we need to properly evaluate the QC electronic ground state energy when including the QC-environment interaction (perturbed electronic ground eigenstate energy). The CT reactions we consider in this paper involve as charge donor and acceptor two Gauanine bases separated by 1–3 in between other bases. Therefore, we should in principle consider as QC the whole chemical complex including the two Guanines as well as the in between other bases, thus requiring to address several difficulties for modeling such a large and flexible QC. However, due to the essentially electronically uncoupled donor and acceptor chemical groups (i.e., the two Guanines), we can evaluate the electronic transition energy $\Delta U_{e}\left(\xi_{P},\beta_{P}\right)=U_{e}\left(\xi_{P},\beta_{P}\right)-U_{e}\left(\xi_{R},\beta_{R}\right)$ approximating the whole transition energy (i.e., $\Delta U_{e}\left(\xi_{P},\beta_{P}\right)\approx \Delta U$, see Equation (27)) and thus using it to evaluate the diabatic energy crossing (i.e., ΔU=0), by treating the acceptor and donor groups as two electronically independent QC’s each perturbed by its environment [37]. In fact, within such an approximation we can obtain the transition energy of the CT process via either the reaction scheme I

(32) $$e^{-}+D^{+}+A\to D+A\to D+A^{+}+e^{-}$$

or the equivalent reaction scheme II

(33) $$D^{+}+A\to D^{+}+A^{+}+e^{-}\to D+A^{+}$$

where $e^{-}$ is the electron formally added or subtracted in the process, $D^{+}$ and *D* are the charged and neutral hole donor species, $A^{+}$ and *A* are the charged and neutral hole acceptor species, in the first step of scheme I as well as in the second step of scheme II, the QC is the donor only (i.e., *A* and $A^{+}$ are treated as part of the perturbing environment) and in the second step of scheme I as well as in the first step of scheme II, the QC is the acceptor only (i.e., *D* and $D^{+}$ are treated as part of the perturbating environment).

Therefore, we can write

(34) $$U_{e}\left(\xi_{P},\beta_{P}\right)-U_{e}\left(\xi_{R},\beta_{R}\right)≅\Delta U_{e,D^{+},red}^{I}+\Delta U_{e,A,ox}^{I}≅\Delta U_{e,A,ox}^{II}+\Delta U_{e,D^{+},red}^{II}$$

where $\Delta U_{e,D^{+},red}^{I},\Delta U_{e,D^{+},red}^{II}$ are the donor species perturbed electronic ground state energy change due to reduction in the reaction steps $e^{-}+D^{+}+A\to D+A$ and $D^{+}+A^{+}+e^{-}\to D+A^{+}$, respectively, and $\Delta U_{e,A,ox}^{I},\Delta U_{e,A,ox}^{II}$ are the acceptor species perturbed electronic ground state energy change due to oxydation in the reaction steps $D+A\to D+A^{+}+e^{-}$ and $D^{+}+A\to D^{+}+A^{+}+e^{-}$, respectively.

We obtained the perturbed electronic ground state energy at each Molecular Dynamics (MD) simulation frame for each of the QC considered by means of the Perturbed Matrix Method (PMM) [43,44,45] diagonalizing at each MD step the QC electronic Hamiltonian matrix given by the elements [45]

(35) $$H_{e,l,l^{\prime }}≅\delta_{l,l^{\prime }}\left[U_{e,l}^{0}+\sum_{n}V_{n}q_{n,l}^{0}\right]-\left(1-\delta_{l,l^{\prime }}\right)E_{G}\cdot \mu_{e,l,l^{\prime }}^{0}+\delta_{l,l^{\prime }}\Delta V$$

where $U_{e,l}^{0}$ is the *l*th unperturbed electronic state energy, *n* runs over all the QC atoms, $V_{n}$ is the perturbing electric potential at the *n* atom position, $E_{G}$ is the perturbing electric field at the QC centre of mass, $q_{n,l}^{0}$ is the *n* atom charge provided by the *l*th unperturbed electronic state, $\mu_{e,l,l^{\prime }}^{0}$ is the QC unperturbed $l,l^{\prime }$ electronic dipole and ΔV approximates all the higher order terms as a short range potential depending only on the nuclear positions (i.e., identical for all the matrix elements).

**Figure 2:**
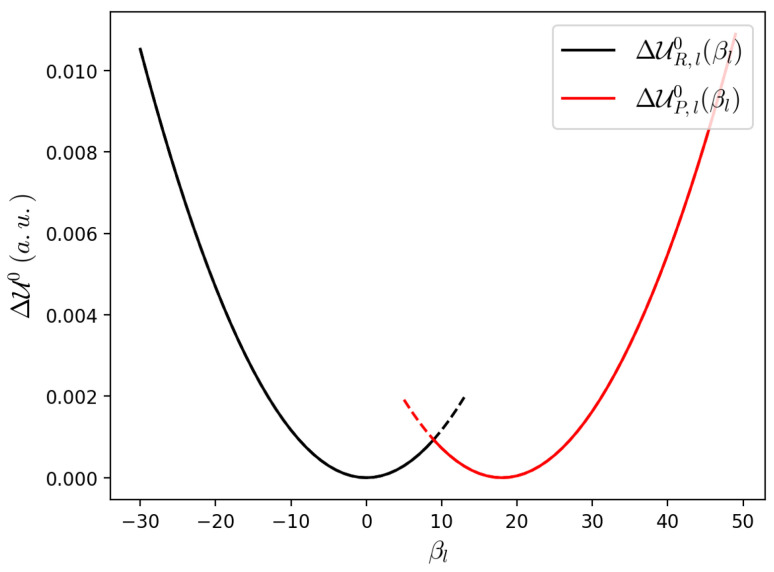
*R* and *P* unperturbed harmonic energies. After the intersection $\Delta U_{R,l}^{0}$ is represented as a black dashed line and before the intersection $\Delta U_{P,l}^{0}$ is represented as a red dashed line.

Finally, considering that the perturbation can only provide slight variations of the vibrational wavefunctions (i.e., the perturbed vibrational eigenstates) we used the unperturbed harmonic wavefunctions of the *R* and *P* minima (i.e., the unperturbed harmonic vibrational eigenstates as obtained by the gas-state donor-acceptor complex, including the in between bases) to obtain the R,P coupling at the crossing, according to Equation (31) (we used the thermal energy for discriminating between semiclassical and quantum modes, with the quantum modes being those corresponding to an energy gap higher than the thermal energy). Moreover, considering also that from Equation (30) we assume the electronic energy change along each quantum mode as independent of the other coordinates position, we approximated the electronic energy change along each *l* quantum mode, $\Delta U_{e}\left(\xi_{R},\beta_{R}^{\prime },\beta_{l}\right)$, by the corresponding *R* and *P* unperturbed harmonic energies ($\Delta U_{R,l}^{0}$ and $\Delta U_{P,l}^{0}$, respectively): switching from $\Delta U_{R,l}^{0}$ to $\Delta U_{P,l}^{0}$ at the intersection of these two unperturbed harmonic energy curves, as shown in Figure 2. Note that the approximation of using the unperturbed vibrational eigenstates and frequencies for evaluating the diabatic state coupling, means that we assume negligible perturbation effects on $H_{R,P}$, and thus for a rigid or semirigid QC (the case studied in this paper) a single unperturbed QC structure calculation is needed to evaluate the transmission coefficient α.

## 3. Computational Details

### 3.1. Computational Strategy

The MD-PMM procedure is at the basis of the whole work and requires the production of MD simulations of the entire system and quantum-chemical calculations on the isolated region of the system selected as the quantum center (QC). In the present case, the Guanine base, is selected as a semi-rigid QC in each investigated system. Two Guanine bases, one (neutral) acting as an electron donor and one (cationic) as an electron acceptor, were selected in each considered double strand. One step is the simulation of the solvated double strand that presents an electronic hole in correspondence of a Guanine site. The double strand sequences were selected from the work of Takada et al. [8] to provide a comparison with experimental data and are the following: ds-5${}^{\prime }$-d(AAAAAAG${}_{1}$AG${}_{2}$XG${}_{3}$AG${}_{4}$A)-3${}^{\prime }$, where X={A,AA,AAA,T,TT,TTT} is the bridge of nucleobases between $G_{2}$ and $G_{3}$. The electron hole was simulated in correspondence of the second Guanine base in the double strand, i.e. $G_{2}^{\cdot +}$ is the electron acceptor and $G_{3}$ is considered as the electron donor. Hence, the considered reactions are the intrastrand electron transfer between $G_{2}^{\cdot +}$ (electron acceptor) and $G_{3}$ (donor) in substrates with different bridge type and length, X, and the rate constants for this process were calculated using scheme I (32).

Additional simulations of solvated double strands with sequence: ds-5${}^{\prime }$-d(AAAAAAG${}_{1}$AG${}_{2}$XC${}_{3}$AC${}_{4}$A)-3${}^{\prime }$, with X={A,T}, were conducted in order to evaluate the intermolecular rate constants of the electron transfer process between $G_{2}^{\cdot +}$ (electron acceptor) and the complementary Guanine base of $C_{3}$ (donor), again using scheme I (Equation (32)).

The other step is the quantum chemical calculations performed on the QC in each redox state, i.e., Guanine base in neutral and radical cation state, in order to apply the reaction scheme in the MD-PMM framework.

After the evaluation of the QC unperturbed properties and the substrates MD simulations, it is possible to apply the MD-PMM procedure to obtain the electronic transition energy distribution and performing the evaluation of the charge transfer rate constant (via Equation (19)).

The calculation of the hessian matrices of the structures of the acceptor-bridge-donor base stack, $G_{2}{\mathrm{XG}}_{3}$, where $G_{2}$ is in the equilibrium geometry of cationic Guanine and $G_{3}$ is in the equilibrium geometry of the neutral Guanine (*R*) and vice versa (*P*), are necessary for the estimation of the R,P coupling. From the hessian matrices, the ground state vibrational eigenstates of *R* and *P* and the frequencies of the quantum vibrational modes are obtained. From single point calculations along the selected eigenvectors, the electronic energy change, with respect to the *R* and *P* minimum are obtained and necessary for the evaluation of the coupling term at crossing expressed by Equation (31).

### 3.2. Molecular Dynamics Simulations

All simulations were carried out using Gromacs software package [46] and AMBER99 force field [47]. The initial structures of the double strands were built in an ideal B-DNA conformation. For the atomic charges of the hole donor $G_{2}^{.+}$ in the double strands we used the ESP charges [48] as obtained for the isolated (gas-phase) radical cation Guanine base, employing the same procedure utilized in the AMBER force field [47] and hence we modified accordingly the original force field to model the reactant state ensemble. Double strands were simulated within a cubic box with edge of 7.95 nm filled with 16490 SPC [49] (simple point charges) water molecules and a number of ${\mathrm{Na}}^{+}$ ions to achieve the system electroneutrality. The velocity rescaling algorithm [50] was used to keep the temperature constant at 300 K. The simulations lasted 100 ns and we used a time step of 2 fs. The volume of the simulation box was fixed, with the MD simulation providing the NVT ensemble statistics.

Additional MD simulations using the BSC1 [51] force field and TIP3P [52] water model were conducted (details in Supplementary Materials).

### 3.3. Unperturbed Quantum States and Properties

The electronic structure calculations were performed using Gaussian 16 [53] software package. The geometries of Guanine base in neutral and radical cation state in gas phase were optimized with density functional theory (DFT) at CAM-B3LYP [54,55]/6-311++G(2d,2p) [56,57] level of theory. The unperturbed electronic properties (energies, electric dipole moments, atomic charges) of the electronic ground and first six excited states were calculated at the DFT and time dependent-DFT (TD-DFT) level of theory using the CAM-B3LYP functional and the 6-311++G(2d,2p) basis set.

The acceptor-bridge-donor stacks used to evaluate the *R* and *P* mass-weighted Hessians were constructed using the optimized geometries of the single bases (at DFT/CAM-B3LYP level of theory), placing them in the conformation of an ideal B-DNA. For the vibrational frequency calculations on the acceptor-bridge-donor stacks, we again made use of DFT at CAM-B3LYP/6-31G(d) [57,58,59] level of theory.

Calculations of the partial atomic charges (ESP charges) for the acceptor-bridge-donor stack, at DFT/CAM-B3LYP and MP2 level of theory, with 6-31G(d) basis set, were made for checking the (positive) charge localization on the single Guanine base.

## 4. Results and Discussion

In line with the available literature, we assumed the relevant hole transfer reaction in DNA molecules as corresponding to the charge transfer between Guanines, even when separated by in between bridge bases (superexchange process): each hole transfer step involves a single donor-bridge-acceptor unit, with the Guanine bases acting as hole donor and acceptor. Note that we disregard the possible involvement of proton transfer in the CT reaction mechanism, as the only evidence for such an effect in the DNA sequences we studied, based on a kinetic isotope-effect experiment, provided the suggestion of only a slight effect on the hole transfer kinetics [8] which is beyond the resolution of our data. The rate constants $K_{CT}$ for the CT reactions between the electron donor (neutral) Guanine base (i.e., the hole acceptor) and the electron acceptor (cationic) Guanine base (i.e., the hole donor) were calculated by means of the MD-PMM procedure as described in the Theory section and compared to the available experimental data as obtained by time-resolved spectroscopy measurements [8].

Firstly, the possible charge delocalization among nucleobases was estimated calculating the atomic charges in different DNA base triplets in cationic form, i.e., (GAG)${}^{+}$ and (GTG)${}^{+}$. To this end, electronic structure calculations of these (gas-phase) DNA triplets in the typical B-DNA geometry at different levels of theory, e.g. MP2 and DFT, were performed. For each base triplet, two sets of nucleobase atomic charges were estimated: one with the $5^{\prime }$ Guanine in its neutral relaxed geometry and the 3${}^{\prime }$ Guanine in its cationic relaxed geometry (the *P* relaxed structure) and the other where such geometries were swapped (the *R* relaxed structure). The results, showing an almost complete localization of the positive charge on a single Guanine (see Table 1) and in agreement with previous computational and experimental works [60,61,62], allowed us to treat the charge transfer process as a charge hopping between two basically electronically independent Guanines as provided (via the super exchange mechanism) by the transition between two energy minima of the electronic ground state of the donor-bridge-acceptor system, in line with the model described in the Theory section (when including in such calculations the SMD implicit solvent model [63] for mimicking the mean solvent effect and the GD3 empirical dispersion [64] we obtain an even stronger charge localization, see Supplementary Materials). It is worth to remark that the use of *R* and *P* diabatic vibrational states each essentially corresponding to a single (ground state) electronic energy minimum with the excess charge localized on one of the two Guanine bases, does not avoid, due to the TR crossing (see Figure 1), the occurrence of charge delocalization (possibly involving also the bridge bases) as suggested by experimental data [65,66] and computational results [16,27]. In fact, within the TR it is expected that the dynamical quantum state of the reaction center (the donor-bridge-acceptor system) become a mixture of the two diabatic states, thus providing charge delocalization. Note that we tested the first electronic excitation as a possible alternative CT reaction channel. However, in the excited electronic state we found an incomplete charge transfer involving very high transition energies (i.e., very slow reaction rate), thus preventing any efficient CT reaction process.

**Figure 3:**
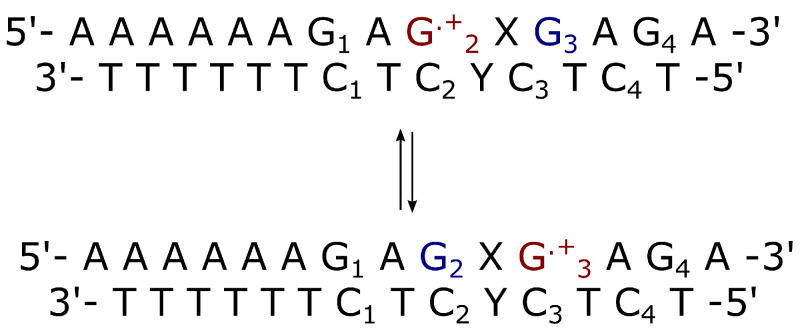
Schematic representation of intrastrand charge transfer in double strands where the bridge is X={A,AA,AAA,T,TT,TTT} and Y is the complementary strand of X. The electron donor is $G_{3}$ (in blue) and the acceptor is $G_{2}^{.+}$ (in red). These two Guanine bases belong to the same strand.

**Figure 4:**
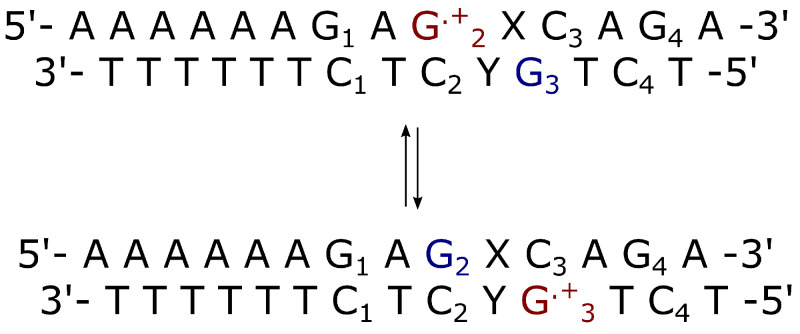
Schematic representation of interstrand charge transfer in double strands where the bridge is X={A,T} and Y is the complementary strand of X. The electron donor is $G_{3}$ (in blue) and the acceptor is $G_{2}^{.+}$ (in red). These two Guanine bases belong to different strands.

**Figure A2:**
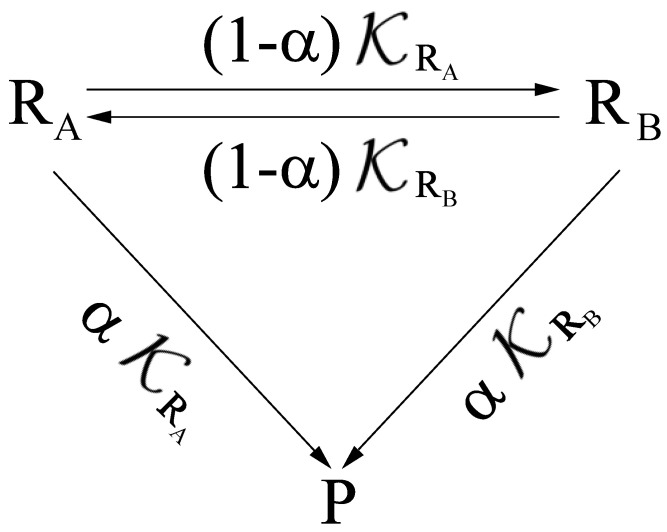
Reaction scheme for the complete R→P transition as occurring through all the TR reactions (see text).

In the CT reaction investigated in this paper, from preliminary PMM-MD calculations, the $R_{A}$ chemical state (the lower energy $R_{I}$ state in Figure 1) is the initial reactant condition with then the $R_{B}$ population (the population of the higher energy $R_{II}$ state in Figure 1) within the steady state approximation. To evaluate the charge transfer rate constant $K_{CT}≅\alpha \left(2-\alpha \right)K_{R_{A}}$ (see Equations (1)–(4) and the reaction scheme of Figure A2) as a function of the length and type of the bridge between the charge donor and acceptor, different double stranded DNA sequences reported in Figure 3 and Figure 4, were considered. Note that due to the slow CT kinetics we always used the Gaussian approximation (see the Theory section) to estimate the kinetic constant $K_{R_{A}}$.

**Figure 5:**
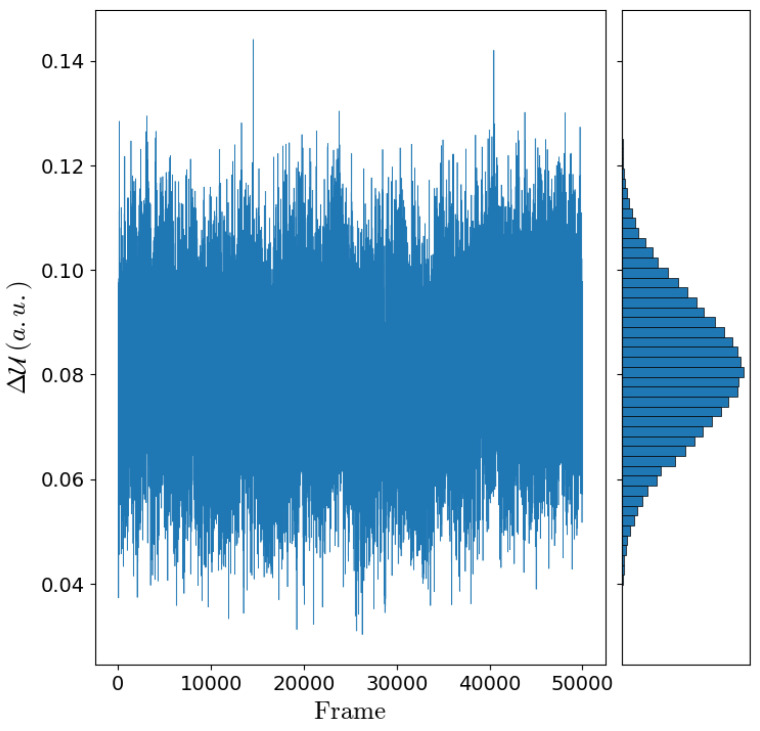
Trajectory and distribution of the electronic transition energies along the MD simulation and its Gaussian behaviour for the ds-5${}^{\prime }$-d(AAAAAAG${}_{1}$AG${}_{2}$TG${}_{3}$AG${}_{4}$A)-3${}^{\prime }$ system.

According to the reaction scheme I (Equation (32)), the electronic transition energies for each DNA molecule were estimated by means of the MD-PMM procedure (see the Theory section). Note that the use of the other, within our approximations, equivalent reaction scheme (reaction scheme II Equation (33)) provided almost identical electronic transition energies and (within the noise) the same results, thus showing the reliability of the approximations employed. In Figure 5, the electronic transition energies and its Gaussian behavior is reported for the ds-5${}^{\prime }$-d(AAAAAAG${}_{1}$AG${}_{2}$TG${}_{3}$AG${}_{4}$A)-3${}^{\prime }$ as an example.

According to Equation (27), from the mean and the variance of the electronic transition energy distribution by means of Equation (19) we calculated the kinetic constant $K_{R_{A}}$ for the intrastrand and interstrand hole transfer between $G_{2}^{.+}$ and $G_{3}$ in the ds-5${}^{\prime }$-d(AAAAAAG${}_{1}$AG${}_{2}$XG${}_{3}$AG${}_{4}$A)-3${}^{\prime }$ substrates (see Table 2 and Table 3).

**Figure 6:**
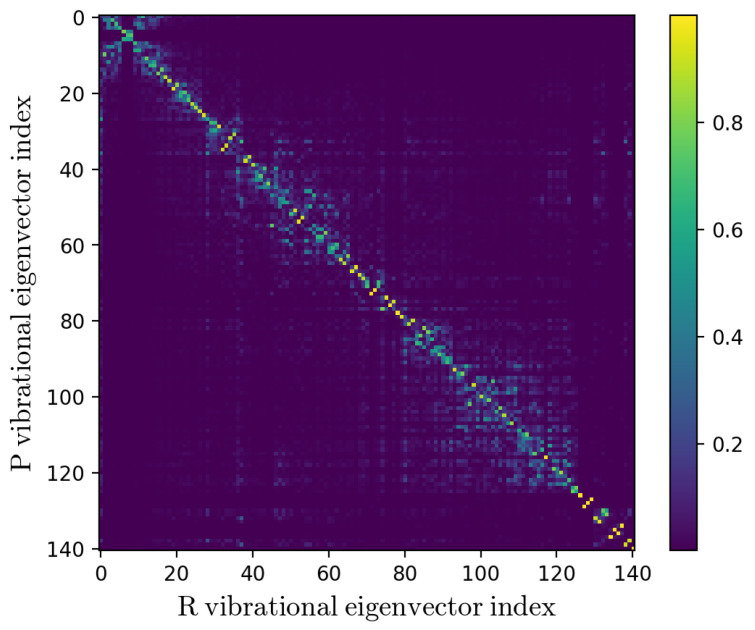
Squared elements of the Duschinsky matrix for the *R* and *P* vibrational eigenvectors (i.e., the *R* and *P* mass-weighted Hessian eigenvectors).

As expected, the kinetic constant $K_{R_{A}}$ for the intrastrand hole transfer between $G_{2}^{.+}$ and $G_{3}$ decreases as the length of the bridge is increasing. This is observed for both the A-type and T-type bridges (see Table 2), in line with the experimental data [8]. Interestingly, the obtained $K_{R_{A}}$ are always close or slower than the experimental rate constants thus suggesting that no relevant diabatic effects are present and hence $K_{CT}≅\lim_{\alpha \to 1}\alpha \left(2-\alpha \right)K_{R_{A}}=K_{R_{A}}$. In order to verify the reliability of the α→1 hypothesis, the transmission coefficient α for the hole transfer process in the substrates containing the sequences $G_{2}{\mathrm{AG}}_{3}$ and $G_{2}{\mathrm{TG}}_{3}$ was estimated, according to the Theory section. For both cases, we considered the (unperturbed) QC formed by the acceptor/donor Guanines and the bridge (the A or T base) in between, calculating the mass-weighted Hessian matrix of the reactant and product electronic ground state energy minimum. By inspecting the squared elements of the Duschinsky matrix (the matrix obtained by the inner products between the *R* and *P* mass-weighted Hessian eigenvectors) for ${\left(\mathrm{GAG}\right)}^{+}$ triplet reported in Figure 6, we can safely conclude that no relevant mixing of the quantum modes is provided by the reactant to product transition, thus allowing us to consider each *R* quantum vibrational mode as essentially coinciding with a *P* mode of virtually identical frequency, with only a significant shift of the minimum energy position (note that the *R* and *P* modes with identical eigenvector index have virtually identical frequencies).

The electronic energy change along the relevant quantum modes were then obtained and hence, via Equation (31) and the Landau-Zener approximation, the value of α≈0.8 and α(2−α)≈0.96 for both triplets were estimated. This clearly confirms that for the $G_{2}{\mathrm{AG}}_{3}$ and $G_{2}{\mathrm{TG}}_{3}$ sequences the investigated hole transfer is essentially adiabatic and hence, for sake of simplicity, we assumed $K_{CT}≅K_{R_{A}}$ as a reasonable approximation for all the sequences considered. Similar calculations for larger QC involving longer bridges are computationally very demanding and possibly inaccurate due to the complexity of the system and the large dimension of the Hessian to be used.

**Figure 7:**
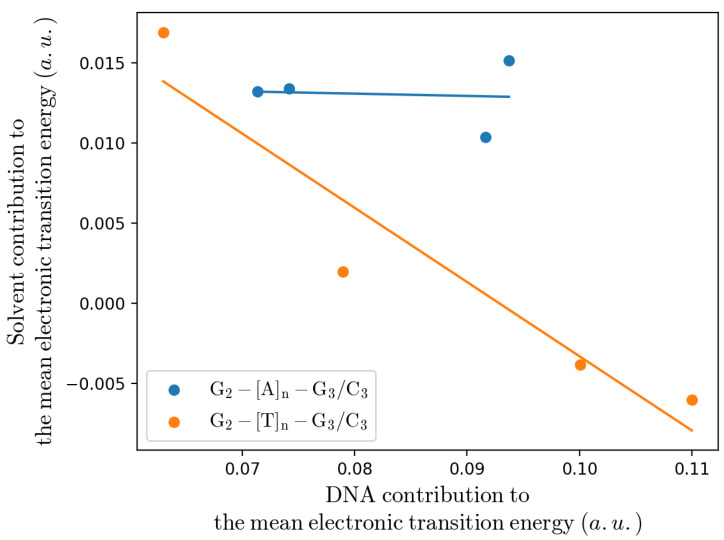
Solvent contribution to the mean electronic transition energy versus the DNA contribution to the mean electronic transition energy. Note that larger DNA contributions correspond to larger bridge lengths and in the T-type bridge systems only for the inter-strand CT the solvent contribution (the highest value) is comparable to the solvent contributions of the A-type bridge systems. The solid lines are the linear regressions of the data.

**Figure 8:**
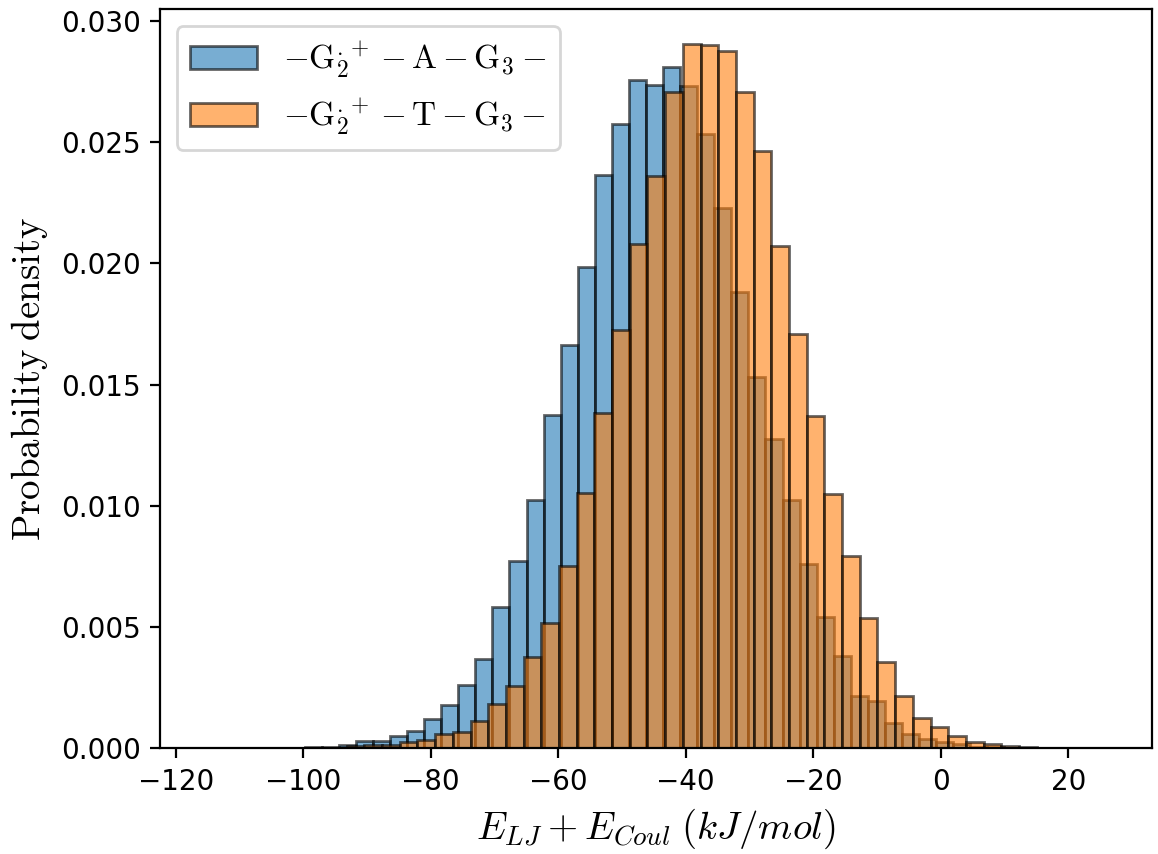
Solvent-$G_{2}^{.+}$ long range interaction energy distributions.

Remarkably, from Table 2 it is evident that our estimate of the (intra-strand) rate constant (i.e., $K_{CT}≅K_{R_{A}}$) for the T-type bridge systems well matches the experimental value for all the sequences, irrespective of the bridge length, while for the A-type bridge systems our calculations always underestimate the rate constant and, in particular, for the single Adenine bridge case a large discrepancy is present. Interestingly, when dissecting the mean electronic transition energy $\langle \Delta U_{e}\left(\xi_{P},\beta_{P}\right)\rangle =\langle \Delta U_{e,D^{+},red}^{I}+\Delta U_{e,A,ox}^{I}\rangle$ into the solvent $\langle \Delta U_{e,solv}\left(\xi_{P},\beta_{P}\right)\rangle$ and DNA $\langle \Delta U_{e,helix}\left(\xi_{P},\beta_{P}\right)\rangle$ contributions (i.e., as obtained considering either only the solvent or only the DNA perturbation), it emerges that for the intra-strand CT the main difference between the A-type and T-type bridge systems is the solvent contribution being much larger in the A-type bridge systems (in the T-type bridge systems an almost null solvent contribution is present, see Supplementary Materials, thus indicating an almost identical solvent average perturbation for $G_{2}^{.+}$ and $G_{3}$) with a clear negative correlation only for the T-type bridge systems (for the A-type bridge systems the solvent contribution seems basically independent of the DNA one, see Figure 7). Such results suggest a possible MD force field inaccuracy for treating the interaction between the solvent and $G_{2}^{.+}$–${\left[A\right]}_{n}$–$G_{3}$, providing a too high solvent contribution to the mean transition energy: e.g., the lack of a proper stacking term between the oxidized Guanine and the Adenine bases could provide an excessive solvent-$G_{2}^{.+}$ interaction in the *R* ensemble, over-stabilizing $G_{2}^{.+}$ as suggested by the comparison of the solvent-$G_{2}^{.+}$ interaction energy distributions as obtained by the MD simulations of the $G_{2}^{.+}$–A–$G_{3}$ and $G_{2}^{.+}$–T–$G_{3}$ sequence containing substrates (see Figure 8). Note that in order to test other force fields, we performed the same calculations using the BSC1 [51] force field or the TIP3P [52] water model finding (within the noise) identical results, see Supplementary Materials.

**Figure 9:**
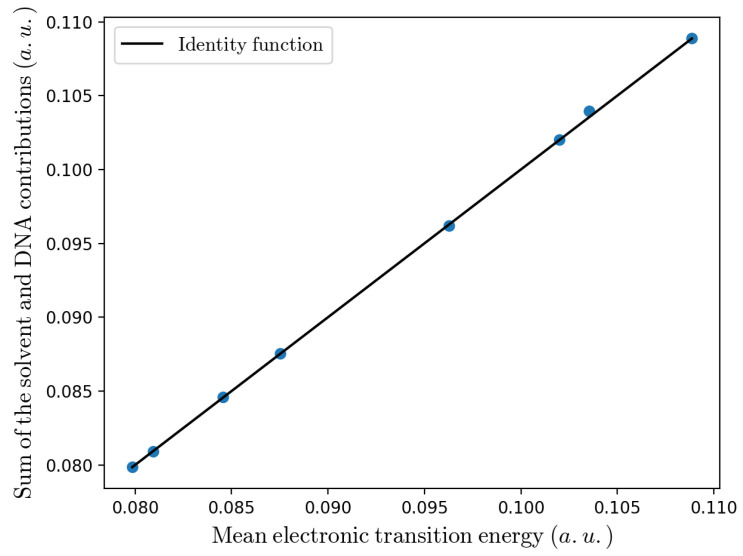
Comparison between the mean electronic transition energy and the sum of the solvent and DNA contributions. The solid line is the bisector of the plane.

Considering that in the present study we deal with the electronic ground state energy and thus our PMM calculations are basically equivalent to the first order perturbation theory and also realizing that within our reaction model we have null unperturbed electronic transition energy (i.e., $\Delta U_{e}^{0}\left(\xi_{P},\beta_{P}\right)=0$), it follows $\langle \Delta U_{e}\left(\xi_{P},\beta_{P}\right)\rangle ≅\langle \Delta U_{e,solv}\left(\xi_{P},\beta_{P}\right)\rangle +\langle \Delta U_{e,helix}\left(\xi_{P},\beta_{P}\right)\rangle$ (see Figure 9) clearly allowing us to roughly simulate the effect of a reduced solvent-$G_{2}^{.+}$ interaction for the A-type bridges by simply partly or fully removing from the mean electronic transition energy the solvent contribution (we assume that both the DNA contribution as well as the overall electronic transition energy variance be always properly provided by the MD simulations). In Table 4 we show the calculated kinetic constants for the $G_{2}^{.+}$–${\left[A\right]}_{n}$–$G_{3}$ sequence containing substrates, when removing/reducing the solvent contribution to the mean electronic transition energy. From the Table it is indeed evident that when using such a rough correction we can obtain a good reproduction of the experimental CT rate constants, with a clear indication that the over-estimation of the solvent contribution reduces as the A-type bridge becomes larger and the two Guanines are subjected to an increasingly different average solvent perturbation field. Similar results are obtained when the electron acceptor $G_{2}^{.+}$ and the electron donor $G_{3}$ are on different strands as for the $G_{2}^{.+}$–A–$C_{3}$ and $G_{2}^{.+}$–T–$C_{3}$ sequence containing substrates (see Table 3 and Table 5). Again the CT rate constant is properly obtained for the T-type bridge system (although now the solvent contribution to the mean electronic transition energy is relevantly larger than zero), while for the A-type bridge system a reasonable reproduction of the experimental rate constant can be obtained only when reducing the solvent contribution to the mean electronic transition energy. It is worth noting that, differently from the intrastrand CT case, the single Adenine bridge system requires a reduction of the solvent contribution instead of a full removal, as expected from the larger difference of the average perturbation fields experienced by the two Guanines on the different strands, in line with what observed for the intrastrand CT in the largest A-type bridge system.

**Figure 10:**
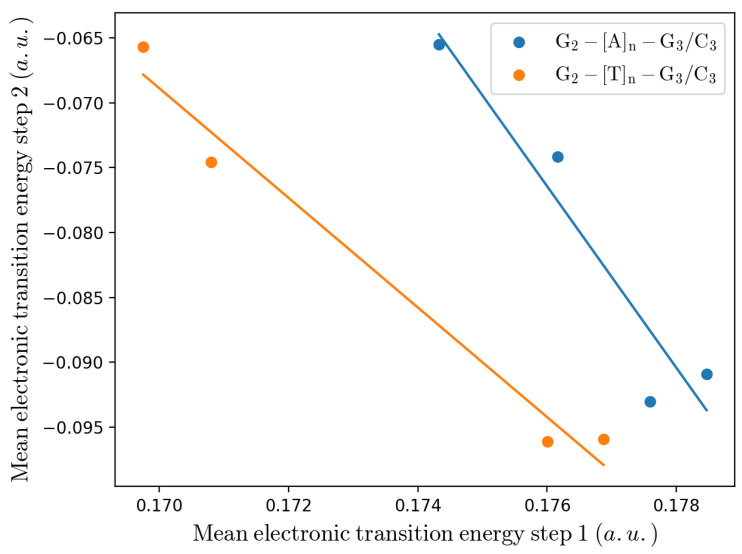
Mean electronic transition energy of the first reaction step of scheme I (subtracted of the corresponding unperturbed electronic transition energy), versus the mean electronic transition energy of the second reaction step of scheme I (subtracted of the corresponding unperturbed electronic transition energy). Note that larger first step mean transition energies correspond to larger bridge lengths. The solid lines are the linear regressions of the data.

In order to better understand the mechanism determining the CT reaction studied, we compared the mean electronic transition energies of the two steps of the reaction scheme I, as obtained for the A-type and T-type bridge systems. A detailed analysis of these single step mean transition energies, also dissecting them into the solvent and DNA contributions (see Supplementary Materials), showed for both systems a similar behavior: the overall perturbation (DNA plus solvent) provides in the *R* ensemble a relevant stabilization of the charged Guanines ($G_{2}^{.+}$ in the first reaction step and $G_{3}^{.+}$ in the second reaction step), as shown by the positive mean electronic transition energy in the first step $\langle \Delta U_{e,1}\left(\xi_{P},\beta_{P}\right)\rangle =\langle \Delta U_{e,D^{+},red}^{I}\rangle$ (once subtracted of the corresponding unperturbed electronic transition energy $\Delta U_{e,1}^{0}\left(\xi_{P},\beta_{P}\right)=-0.2796$ a.u.) and the negative mean transition energy in the second step $\langle \Delta U_{e,2}\left(\xi_{P},\beta_{P}\right)\rangle =\langle \Delta U_{e,A,ox}^{I}\rangle$ (once subtracted of the corresponding unperturbed electronic transition energy $\Delta U_{e,2}^{0}\left(\xi_{P},\beta_{P}\right)=0.2796$ a.u.), resulting in an increasing preferential stabilization of $G_{2}^{.+}$ (i.e., $\langle \Delta U_{e}\left(\xi_{P},\beta_{P}\right)\rangle =\langle \Delta U_{e,1}\left(\xi_{P},\beta_{P}\right)\rangle +\langle \Delta U_{e,2}\left(\xi_{P},\beta_{P}\right)\rangle >0$) as the bridge length becomes larger and a clear negative correlation between them, see Figure 10 (note again that $\Delta U_{e,1}^{0}\left(\xi_{P},\beta_{P}\right)+\Delta U_{e,2}^{0}\left(\xi_{P},\beta_{P}\right)=\Delta U_{e}^{0}\left(\xi_{P},\beta_{P}\right)=0$). However, while the DNA perturbation effect favors the charged Guanine state (i.e., $\langle \Delta U_{e,helix,1}\left(\xi_{P},\beta_{P}\right)\rangle -\Delta U_{e,1}^{0}\left(\xi_{P},\beta_{P}\right)>0$ and $\langle \Delta U_{e,helix,2}\left(\xi_{P},\beta_{P}\right)\rangle -\Delta U_{e,2}^{0}\left(\xi_{P},\beta_{P}\right)<0$), the solvent perturbation effect favors the neutral Guanine state (i.e., $\langle \Delta U_{e,solv,1}\left(\xi_{P},\beta_{P}\right)\rangle -\Delta U_{e,1}^{0}\left(\xi_{P},\beta_{P}\right)<0$ and $\langle \Delta U_{e,solv,2}\left(\xi_{P},\beta_{P}\right)\rangle -\Delta U_{e,2}^{0}\left(\xi_{P},\beta_{P}\right)>0$), with again a clear negative correlation between such solvent and DNA contributions (see Supplementary Materials).

## 5. Conclusions

In this paper we extend the general model [31] for the kinetics of CT reactions, introducing specific modifications to treat the CT processes as occurring via the hopping between energy minima of the same electronic state (i.e., tunnelling between the corresponding vibrational ground states). In the Theory section we describe the theoretical framework and practical strategy for modelling such CT reactions, deriving and discussing in details the Gaussian approximation. Application of the theoretical–computational model to CT reactions in DNA molecules confirmed the widely shared idea that Guanine bases act as the relevant charge carriers (i.e., hole donor and acceptor), characterizing the CT process for the considered sequences as the charge hopping between subsequent Guanines via the super exchange mechanism [8,17,22,30].

Comparison of our data with the available experimental ones provided several important results:

- The CT reaction within a single hole hopping step can be conceived as the tunnelling between the vibrational ground states of two different electronic ground state energy minima of the donor-bridge-acceptor unit, each characterized by the excess charge located on one of the two Guanine bases involved.
- In modelling the transition energy, the hole donor and acceptor Guanine bases can be treated as essentially electronically independent quantum systems, suggesting that delocalization of the excess charge is not relevant for modelling the kinetics of these reactions. Note that such finding does not avoid the occurrence of charge delocalization due to the TR crossing, see Figure 1.
- The fluctuating perturbation field acting on the Guanine bases, due to the DNA and solvent dynamics, is the driving force of the CT reactions studied, confirming the essential role of the perturbing environment we found in previous works on different chemical systems.
- All the CT reactions investigated can be described as largely adiabatic processes, regardless of the bridge type, in line with our explicit evaluation of the transmission coefficient in the single base bridge systems.
- While for the T-type bridge systems our model provides an accurate reproduction of the experimental reaction rate constants, for the A-type bridge systems significant deviations between the calculated and the experimental rate constants are present, probably due to the inaccuracy of the MD force field in treating the interaction between the solvent and the A-type bridge systems (resulting in an over-stabilized $G_{2}^{.+}$) as suggested by the reasonably accurate rate constants for the A-type bridge systems when the solvent contribution is partly or fully removed from the mean transition energy.
- While the DNA perturbation favors the charged Guanine state, the solvent perturbation favors the neutral Guanine state with the resulting overall perturbation providing a decreasing rate constant (increasing mean transition energy) as the bridge length increases.

Finally, it is worth to remark that the presented theoretical–computational model, specifically designed for CT reactions due to the tunnelling between (diabatic) vibrational states of the same electronic state, could furnish a general proper quantitative description of the CT reactions as occurring without requiring prior electronic excitation, thus involving the electronic ground state only. Therefore, such an approach for treating the hopping between vibrational states of different minima of the electronic ground state together with the previously described method [31] for CT reactions involving tunnelling between different (diabatic) electronic states, can provide a general relatively simple approach to quantitatively describe CT reactions, particularly suited when dealing with complex systems.

## Figures and Tables

**Table 1:** ESP charges of (GAG)${}^{+}$ and (GTG)${}^{+}$ stacks. Two sets of ESP charges for each triplet are reported: one where the $5^{\prime }$ Guanine is in its neutral relaxed geometry and the $3^{\prime }$ Guanine in its cationic relaxed geometry and one where the Guanine geometries were swapped.

Base	q	q
	**CAM-B3LYP**	**MP2**
$5^{\prime }$ $G^{.+}$	0.87	0.90
A	0.07	0.06
$3^{\prime }$ G	0.06	0.04
$5^{\prime }$ $G^{.+}$	0.92	0.94
T	0.02	0.01
$3^{\prime }$ G	0.06	0.04
$5^{\prime }$ G	0.08	0.01
A	0.09	0.06
$3^{\prime }$ $G^{.+}$	0.83	0.93
$5^{\prime }$ G	0.09	0.04
T	0.00	−0.02
$3^{\prime }$ $G^{.+}$	0.91	0.96

**Table 2:** Comparison between the calculated kinetic constant $K_{R_{A}}$ and the experimentally measured rate constant $K_{CT}$ for the intrastrand charge transfer of the $G_{2}^{.+}$– ${\left[A\right]}_{n}$–$G_{3}$ and $G_{2}^{.+}$–${\left[T\right]}_{n}$–$G_{3}$ sequence containing substrates. For all the calculated $K_{R_{A}}$ the relative standard error is about 30 per cent of the estimated kinetic constant.

n	$K_{R_{A}}\left(s^{-1}\right)$	$K_{\mathrm{CT}}\left(s^{-1}\right)$
${\left[A\right]}_{n}$	PMM	exp. [8]
1	$2.8\times 10^{4}$	$4.8\times 10^{7}$
2	$5.2\times 10^{3}$	$9.7\times 10^{4}$
3	$2.0\times 10^{3}$	$1.4\times 10^{4}$
${\left[T\right]}_{n}$		
1	$1.6\times 10^{5}$	$4.6\times 10^{5}$
2	$1.4\times 10^{4}$	$3.6\times 10^{4}$
3	$1.7\times 10^{4}$	$9.1\times 10^{3}$

**Table 3:** Comparison between the calculated kinetic constant $K_{R_{A}}$ and the experimentally measured rate constant $K_{CT}$ for the interstrand charge transfer of the $G_{2}^{.+}$–A–$C_{3}$ and $G_{2}^{.+}$–T–$C_{3}$ sequence containing substrates. In this case, the electron donor, $G_{3}$ Guanine base, is in the complementary strand.

	$K_{R_{A}}\left(s^{-1}\right)$	$K_{\mathrm{CT}}\left(s^{-1}\right)$
	PMM	exp. [8]
A	$1.8\times 10^{5}$	$1.4\times 10^{6}$
T	$2.9\times 10^{6}$	$1.6\times 10^{6}$

**Table 4:** Comparison between the calculated kinetic constant $K_{R_{A}}$ (obtained removing from the mean transition energy the solvent contribution) and the experimentally measured rate constant $K_{CT}$ for the intrastrand charge transfer of the $G_{2}^{.+}$–${\left[A\right]}_{n}$–$G_{3}$ sequence containing substrates. When not specified the data refer to fully removed solvent contribution.

n	$K_{R_{A}}\left(s^{-1}\right)$	$K_{\mathrm{CT}}\left(s^{-1}\right)$
${\left[A\right]}_{n}$	PMM	exp. [8]
1	$9.1\times 10^{6}$	$4.8\times 10^{7}$
2	$3.4\times 10^{5}$	$9.7\times 10^{4}$
2 ^*a*^	$4.5\times 10^{4}$	$9.7\times 10^{4}$
3	$1.2\times 10^{6}$	$1.4\times 10^{4}$
3 ^*a*^	$7.7\times 10^{4}$	$1.4\times 10^{4}$

^*a*^ Value obtained including in the mean transition energy one half of the solvent contribution.

**Table 5:** Comparison between the calculated kinetic constant $K_{R_{A}}$ (obtained removing from the mean transition energy the solvent contribution) and the experimentally measured rate constant $K_{CT}$ for the interstrand charge transfer of the $G_{2}^{.+}$–A–$C_{3}$ sequence containing substrates. In this case, the electron donor, $G_{3}$ Guanine base, is in the complementary strand. When not specified the data refer to fully removed solvent contribution.

	$K_{R_{A}}\left(s^{-1}\right)$	$K_{\mathrm{CT}}\left(s^{-1}\right)$
	PMM	exp. [8]
A	$3.1\times 10^{7}$	$1.4\times 10^{6}$
A ^*a*^	$2.6\times 10^{6}$	$1.4\times 10^{6}$

^*a*^ Value obtained including in the mean transition energy one half of the solvent contribution.

## Data Availability

Data available on request.

## Supplementary Materials

The following supporting information can be downloaded at: https://www.mdpi.com/article/10.3390/molecules27217408/s1. Table S1: Helix; Table S2: Solvent; Figure S1: Electronic transition energies (scaled by their respective mean value) distributions obtained when the solvent or helix perturbing electric field alone is considered; Figure S2: Electronic transition energies (scaled by their respective mean value) distributions obtained when the solvent or helix perturbing electric field alone is considered; Figure S3: Correlation between the electronic transition energy trajectories (scaled by their respective mean value) obtained when the solvent or helix perturbing electric field alone is considered; Figure S4: Correlation between the electronic transition energy trajectories (scaled by their respective mean value) obtained when the solvent or helix perturbing electric field alone is considered; Table S3: Step 1 and 2; Table S4: Step 1 and 2, helix contribution; Table S5: Step 1 and 2, solvent contribution; Table S6: ESP charges of (GAG)${}^{+}$ and (GTG)${}^{+}$ stacks in water solvent (SMD [63] model was used). The GD3 empirical dispersion [64] was included. Two sets of ESP charges for each triplet are reported: one where the $5^{\prime }$ Guanine is in its neutral relaxed geometry and the $3^{\prime }$ Guanine in its cationic relaxed geometry and one where the Guanine geometries were swapped; Table S7: Comparison between the calculated kinetic constants $K_{R_{A}}$ for the intrastrand charge transfer of the $G_{2}^{.+}$–A–$G_{3}$ sequence containing substrates. A comparison between AMBER99 and BSC1 force fields and between SPC and TIP3P water models are given.

## Appendix A

Whenever a diabatic energy crossing occurs, i.e., a TR traversing, a possible reaction event can be present according to the scheme of Figure 1. From the Figure, beyond the TR itself, we can identify four different chemical states ($R_{I},R_{II}$ and $P_{I},P_{II}$) corresponding to perturbation regions where the adiabatic states *I* and II are virtually identical either to the *R* or the *P* diabatic state. According to Figure 1, using the definitions and assumptions previously described, at each TR crossing process the adiabatic fraction χ is provided by the Landau-Zener approximation: i.e., $\chi ≅1-e^{-\frac{2\pi |H_{R,P}{|}^{2}}{\hbar \;v_{cr}}}$ with $H_{R,P}$ the Hamiltonian reactant-product coupling and $v_{cr}$ the absolute value of the diabatic energy difference time derivative at the crossing (i.e., crossing velocity). Defining with *A* and *B* the *I* and II adiabatic surface index, respectively, the reaction scheme for the $R_{A}\to P$ transition as occurring within a single TR reaction (i.e., a reaction process defined by crossing events characterized by almost identical $H_{R,P}$, $v_{cr}$ and hence with an almost identical χ value) is provided by Figure A1.

Therefore, according to the complete reaction scheme (see Figure A2) for the R→P transition as occurring through all the TR reactions (i.e., considering all the crossing events), we can write [31]

(A1) $$\begin{array}{ccc} \dot{\left[R_{A}\right]} & ≅ & -K_{R_{A}}\left[R_{A}\right]+\left(1-\alpha \right)\;K_{R_{B}}\left[R_{B}\right] \end{array}$$

(A2) $$\begin{array}{ccc} \dot{\left[R_{B}\right]} & ≅ & \left(1-\alpha \right)\;K_{R_{A}}\left[R_{A}\right]-K_{R_{B}}\left[R_{B}\right] \end{array}$$

(A3) $$\begin{array}{ccc} \dot{\left[P\right]} & ≅ & -\left(\dot{\left[R_{B}\right]}+\dot{\left[R_{A}\right]}\right)≅\alpha K_{R_{A}}\left[R_{A}\right]+\alpha K_{R_{B}}\left[R_{B}\right] \end{array}$$

where $\alpha ={\langle \chi \rangle }_{A}={\langle \chi \rangle }_{B}=\langle \chi \rangle$ and assuming the stationary state for the TR concentration from Figure A1 we have

(A4) $$\begin{array}{ccc} K_{R_{A}} & ≅ & {\langle \frac{k_{R_{A}}k^{+}}{k^{+}+k^{-}}\rangle }_{A}≅{\langle k_{R_{A}}\rangle }_{A} \end{array}$$

(A5) $$\begin{array}{ccc} K_{R_{B}} & ≅ & {\langle \frac{k_{R_{B}}k^{+}}{k^{+}+k^{-}}\rangle }_{B}≅{\langle k_{R_{B}}\rangle }_{B} \end{array}$$

with the angle brackets meaning the average over all the TR reactions, as obtained by either the $R_{A}\to P$ (*A* subscript) or the $R_{B}\to P$ (*B* subscript) transitions, we assumed χ as statistically independent from $\frac{k_{R_{A}}k^{+}}{k^{+}+k^{-}}$ and $\frac{k_{R_{B}}k^{+}}{k^{+}+k^{-}}$ with invariant average value when obtained via the $R_{A}\to P$ or the $R_{B}\to P$ transitions and we used $k^{-}≅0$ as it follows from the tiny perturbation interval corresponding to each TR, implying that its mean residence time is shorter than the autocorrelation mean time of the crossing velocity [31,41]. Equations (A1)–(A3) can be simplified by assuming the stationary condition either for $R_{B}$ ($R_{A}$ is the proper initial reactant state) or for $R_{A}$ ($R_{B}$ is the proper initial reactant state). In the former case (i.e., $\dot{\left[R_{B}\right]}≅0$ and hence $\left[R_{B}\right]≅\left(1-\alpha \right)\;K_{R_{A}}\left[R_{A}\right]/K_{R_{B}}$) we have

(A6) $$\begin{array}{ccc} \dot{\left[R_{A}\right]} & ≅ & -\alpha \left(2-\alpha \right)K_{R_{A}}\left[R_{A}\right] \end{array}$$

(A7) $$\begin{array}{ccc} \dot{\left[P\right]} & ≅ & -\dot{\left[R_{A}\right]}≅\alpha \left(2-\alpha \right)K_{R_{A}}\left[R_{A}\right] \end{array}$$

readily providing when defining with ${\left[R_{A}\right]}_{0}$ the initial $R_{A}$ species concentration (i.e. at the beginning of the $R_{B}$ stationary state condition) and neglecting the $R_{B}$ concentration (i.e., $\left[P\right]≅{\left[R_{A}\right]}_{0}-\left[R_{A}\right]$),

(A8) $$\begin{array}{ccc} \left[R_{A}\right] & ≅ & {\left[R_{A}\right]}_{0}\;e^{-\alpha \left(2-\alpha \right)K_{R_{A}}t} \end{array}$$

(A9) $$\begin{array}{ccc} \left[P\right] & ≅ & {\left[R_{A}\right]}_{0}\left[1-e^{-\alpha \left(2-\alpha \right)K_{R_{A}}t}\right] \end{array}$$

For the latter case (i.e., $\dot{\left[R_{A}\right]}≅0$ and hence $\left[R_{A}\right]≅\left(1-\alpha \right)\;K_{R_{B}}\left[R_{B}\right]/K_{R_{A}}$) we can write

(A10) $$\begin{array}{ccc} \dot{\left[R_{B}\right]} & ≅ & -\alpha \left(2-\alpha \right)K_{R_{B}}\left[R_{B}\right] \end{array}$$

(A11) $$\begin{array}{ccc} \dot{\left[P\right]} & ≅ & -\dot{\left[R_{B}\right]}≅\alpha \left(2-\alpha \right)K_{R_{B}}\left[R_{B}\right] \end{array}$$

providing

(A12) $$\begin{array}{ccc} \left[R_{B}\right] & ≅ & {\left[R_{B}\right]}_{0}\;e^{-\alpha \left(2-\alpha \right)K_{R_{B}}t} \end{array}$$

(A13) $$\begin{array}{ccc} \left[P\right] & ≅ & {\left[R_{B}\right]}_{0}\left[1-e^{-\alpha \left(2-\alpha \right)K_{R_{B}}t}\right] \end{array}$$

with obviously ${\left[R_{B}\right]}_{0}$ the initial $R_{B}$ species concentration (i.e., at the beginning of the $R_{A}$ stationary state condition) and again neglecting the $R_{A}$ concentration (i.e., $\left[P\right]≅{\left[R_{B}\right]}_{0}-\left[R_{B}\right]$).

Finally, it is worth noting that in the present case where we deal with diabatic states defined via the vibrational states of different minima of the electronic ground state, considering that the *R* diabatic state corresponding to the relevant reactant condition involves only the vibrational ground state, we can disregard all the possible diabatic energy crossings due to the *P* diabatic states involving the excited vibrational states (i.e., highly diabatic crossings), thus assuming as productive crossings only those due to the *R* and *P* diabatic states involving the vibrational ground states (on the contrary when dealing with diabatic states defined via different electronic states several *P* diabatic vibronic states must be considered [31]).
