# Supplementary material for: Modeling Charge Transfer Reactions by Hopping between Electronic Ground State Minima: Application to Hole Transfer between DNA Bases
Source: Molecules. 2022 Nov 1;27(21):7408. doi: 10.3390/molecules27217408 (PMC9654243; doi:10.3390/molecules27217408)
Supplement: Supplementary file 1 [file molecules-27-07408-s001.zip › molecules-1933893-supplementary.pdf]

Article

Supplementary Information: Modeling charge transfer reactions by hopping between electronic ground state minima: application to hole transfer between DNA bases

Alessandro Nicola Nardi [1], Marco D'Abramo [1*] and Andrea Amadei [2*]

[1] Department of Chemistry, Sapienza University, 00185 Rome, Italy
[2] Department of Chemical and Technological Sciences, Tor Vergata University, 00133 Rome, Italy
* Correspondence: marco.dabramo@uniroma1.it (M.D.); andrea.amadei@uniroma2.it (A.A.)

1. The electronic transition energy when DNA or solvent perturbation alone is considered: mean, standard deviation, distributions and correlations

Table S1. Helix

n	$\langle \Delta\mathcal{U}_{e,helix} \rangle (a.u.)$	$\sigma_{helix} (a.u.)$
$[\mathrm{A}]_n$		
1	0.071366	0.013125
2	0.091630	0.016957
3	0.093723	0.018504
1^a	0.074134	0.016200
$[\mathrm{T}]_n$		
1	0.078946	0.013047
2	0.100051	0.016660
3	0.109994	0.017487
1^a	0.062964	0.018546

^{a} Interstrand.

Table S2. Solvent

n	$\langle \Delta\mathcal{U}_{e,solvent} \rangle (a.u.)$	$\sigma_{solvent} (a.u.)$
$[\mathrm{A}]_n$		
1	0.013220	0.017224
2	0.010379	0.021241
3	0.015150	0.023238
1^a	0.013412	0.020066
$[\mathrm{T}]_n$		
1	0.001969	0.016973
2	-0.003840	0.020914
3	-0.006024	0.022348
1^a	0.016899	0.023098

^{a} Interstrand.

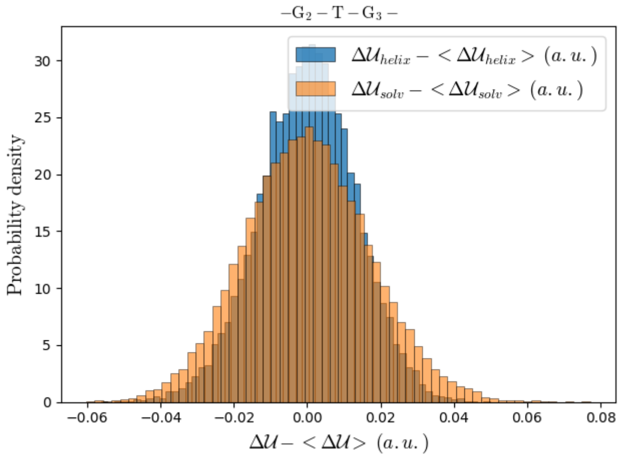

Figure S1. Electronic transition energies (scaled by their respective mean value) distributions obtained when the solvent or helix perturbing electric field alone is considered.

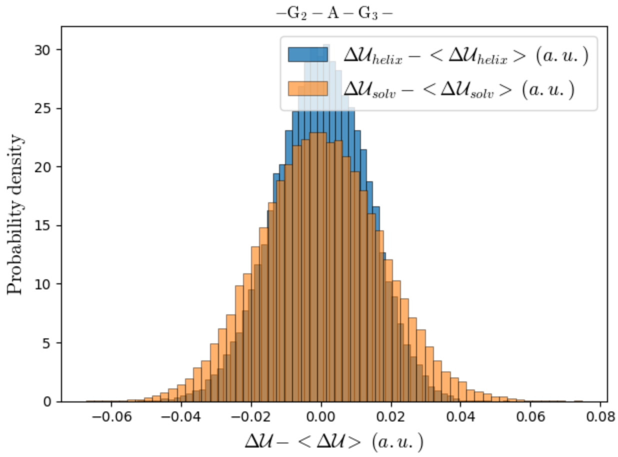

Figure S2. Electronic transition energies (scaled by their respective mean value) distributions obtained when the solvent or helix perturbing electric field alone is considered.

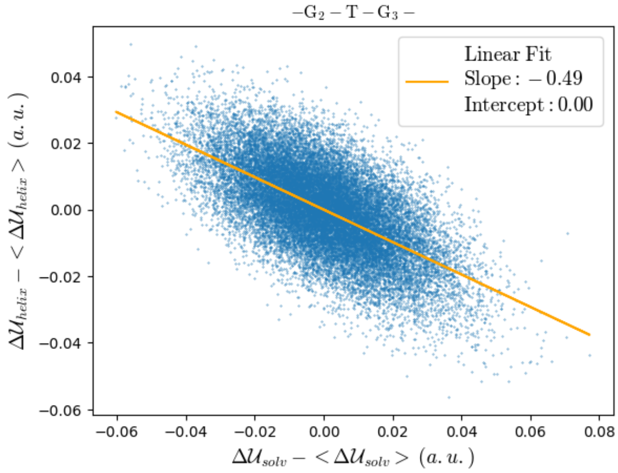

Figure S3. Correlation between the electronic transition energy trajectories (scaled by their respective mean value) obtained when the solvent or helix perturbing electric field alone is considered.

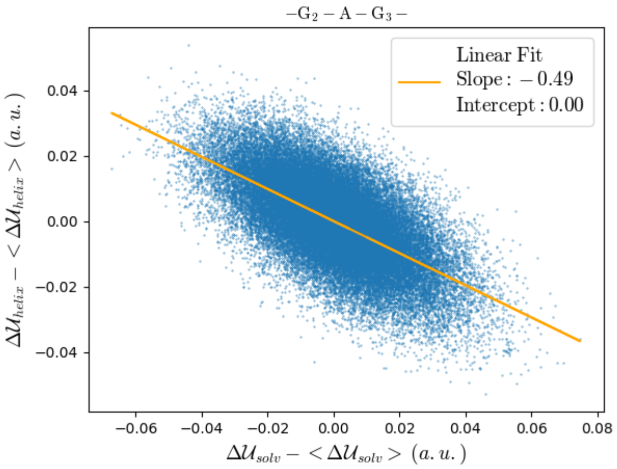

Figure S4. Correlation between the electronic transition energy trajectories (scaled by their respective mean value) obtained when the solvent or helix perturbing electric field alone is considered.

2. Electronic transition energies of step 1 and 2

Table S3. Step 1 and 2.

n	$\langle\Delta\mathcal{U}_{e,1}(a.u.)\rangle$	$\langle\Delta\mathcal{U}_{e,2}\rangle(a.u.)$	$\sigma_1(a.u.)$	$\sigma_2(a.u.)$
$[A]_n$				
1	-0.102081	0.186643	0.012189	0.013883
2	-0.103511	0.205520	0.012268	0.014044
3	-0.105349	0.214212	0.011874	0.014008
1[a]	-0.101209	0.188735	0.012218	0.013657
$[T]_n$				
1	-0.102804	0.183731	0.012258	0.013791
2	-0.108871	0.205134	0.011957	0.013901
3	-0.109923	0.214028	0.011655	0.014074
1[a]	-0.103670	0.183528	0.012328	0.013685

[a] Interstrand.

Table S4. Step 1 and 2, helix contribution.

n	$\langle\Delta\mathcal{U}_{e,helix,1}\rangle(a.u.)$	$\langle\Delta\mathcal{U}_{e,helix,2}\rangle(a.u.)$	$\sigma_{helix,1}(a.u.)$	$\sigma_{helix,2}(a.u.)$
$[A]_n$				
1	0.753346	-0.681980	0.018213	0.015579
2	0.819494	-0.727863	0.019186	0.015687
3	0.844254	-0.750530	0.018203	0.016310
1[a]	0.760421	-0.686287	0.017604	0.020094
$[T]_n$				
1	0.764474	-0.685527	0.016540	0.013764
2	0.815915	-0.715863	0.019697	0.014639
3	0.854893	-0.744898	0.017001	0.013963
1[a]	0.765608	-0.702644	0.018357	0.017191

[a] Interstrand.

Table S5. Step 1 and 2, solvent contribution.

n	$\langle \Delta \mathcal{U}_{e,solv,1} \rangle (a.u.)$	$\langle \Delta \mathcal{U}_{e,solv,2} \rangle (a.u.)$	$\sigma_{solv,1} (a.u.)$	$\sigma_{solv,2} (a.u.)$
$[A]_n$				
1	-1.135103	1.148323	0.020190	0.019512
2	-1.202729	1.213108	0.021119	0.019323
3	-1.244585	1.238182	0.019110	0.019278
1^a	-1.141347	1.154760	0.019901	0.021715
$[T]_n$				
1	-1.147036	1.149006	0.019270	0.018237
2	-1.208250	1.204409	0.021294	0.019079
3	-1.244585	1.238182	0.019110	0.019278
1^a	-1.149028	1.165927	0.021101	0.020008

^{a} Interstrand.

3. ESP charges of $(GAG)^+$ and $(GTG)^+$ triplets including water solvent and dispersion

Table S6. ESP charges of $(GAG)^+$ and $(GTG)^+$ stacks in water solvent (SMD [1] model was used). The GD3 empirical dispersion [2] was included. Two sets of ESP charges for each triplet are reported: one where the 5′ Guanine is in its neutral relaxed geometry and the 3′ Guanine in its cationic relaxed geometry and one where the Guanine geometries were swapped.

Base	q
	CAM-B3LYP/SMD + GD3
5′ $G^{\cdot+}$	0.96
A	0.04
3′ G	0.00
5′ $G^{\cdot+}$	0.99
T	-0.01
3′ G	0.02
5′ G	0.00
A	0.03
3′ $G^{\cdot+}$	0.97
5′ G	0.03
T	-0.03
3′ $G^{\cdot+}$	1.00

4. Calculated kinetic constants for $G_2^{\cdot+}$–A–G_3 sequence containing substrates with different force fields and water models

Additional MD simulations were performed to test other force fields and solvent model, i.e. the BSC1 [3] force field and the TIP3P [4] water model. In Table S7 is reported the comparison between calculated rate constants with the same theoretical-computational method we presented, using BSC1 force field and the TIP3P water model.

Table S7. Comparison between the calculated kinetic constants $\mathcal{K}_{R_A}$ for the intrastrand charge transfer of the $G_2^{\cdot+}$–A–G_3 sequence containing substrates. A comparison between AMBER99 and BSC1 force fields and between SPC and TIP3P water models are given.

	$\mathcal{K}_{R_A} (s^{-1})$
ff	PMM
AMBER99^a	$2.8 \cdot 10^4$
BSC1^a	$1.9 \cdot 10^4$
solv. model	PMM
SPC^b	$2.8 \cdot 10^4$
TIP3P^b	$5.2 \cdot 10^4$

^{a} The SPC water model was used. ^{b} The AMBER99 force field was used.

References

1. Marenich, A.V.; Cramer, C.J.; Truhlar, D.G. Universal solvation model based on solute electron density and on a continuum model of the solvent defined by the bulk dielectric constant and atomic surface tensions. *J. Phys. Chem. B* **2009**, *113*, 6378–6396.
2. Grimme, S.; Antony, J.; Ehrlich, S.; Krieg, H. A consistent and accurate ab initio parametrization of density functional dispersion correction (DFT-D) for the 94 elements H-Pu. *J. Chem. Phys.* **2010**, *132*, 154104.
3. Ivani, I.; Dans, P.D.; Noy, A.; Pérez, A.; Faustino, I.; Hospital, A.; Walther, J.; Andrio, P.; Goñi, R.; Balaceanu, A.; et al. Parmbsc1: a refined force field for DNA simulations. *Nat. Methods* **2016**, *13*, 55–58.
4. Jorgensen, W.L.; Chandrasekhar, J.; Madura, J.D.; Impey, R.W.; Klein, M.L. Comparison of simple potential functions for simulating liquid water. *J. Chem. Phys.* **1983**, *79*, 926–935.
